# Acute phase reactants add little to composite disease activity indices for rheumatoid arthritis: validation of a clinical activity score

**DOI:** 10.1186/ar1740

**Published:** 2005-04-07

**Authors:** Daniel Aletaha, Valerie PK Nell, Tanja Stamm, Martin Uffmann, Stephan Pflugbeil, Klaus Machold, Josef S Smolen

**Affiliations:** 1 Department of Rheumatology, Medical University of Vienna, Vienna, Austria; 2 National Institute of Arthritis and Musculoskeletal and Skin Diseases, National Institutes of Health, Bethesda, Maryland, USA; 3 Department of Radiology, Medical University of Vienna, Vienna, Austria; 4 2nd Department of Medicine, Lainz Hospital, Vienna, Austria

## Abstract

**Introduction:**

Frequent assessments of rheumatoid arthritis (RA) disease activity allow timely adaptation of therapy, which is essential in preventing disease progression. However, values of acute phase reactants (APRs) are needed to calculate current composite activity indices, such as the Disease Activity Score (DAS)28, the DAS28-CRP (i.e. the DAS28 using C-reactive protein instead of erythrocyte sedimentation rate) and the Simplified Disease Activity Index (SDAI). We hypothesized that APRs make limited contribution to the SDAI, and that an SDAI-modification eliminating APRs – termed the Clinical Disease Activity Index (CDAI; i.e. the sum of tender and swollen joint counts [28 joints] and patient and physician global assessments [in cm]) – would have comparable validity in clinical cohorts.

**Method:**

Data sources comprised an observational cohort of 767 RA patients (average disease duration 8.1 ± 10.6 years), and an independent inception cohort of 106 patients (disease duration 11.5 ± 12.5 weeks) who were followed prospectively.

**Results:**

Our clinically based hypothesis was statistically supported: APRs accounted only for 15% of the DAS28, and for 5% of the SDAI and the DAS28-CRP. In both cohorts the CDAI correlated strongly with DAS28 (R = 0.89–0.90) and comparably to the correlation of SDAI with DAS28 (R = 0.90–0.91). In additional analyses, the CDAI when compared to the SDAI and the DAS28 agreed with a weighted kappa of 0.70 and 0.79, respectively, and comparably to the agreement between DAS28 and DAS28-CRP. All three scores correlated similarly with Health Assessment Questionnaire (HAQ) scores (R = 0.45–0.47). The average changes in all scores were greater in patients with better American College of Rheumatology response (*P *< 0.0001, analysis of variance; discriminant validity). All scores exhibited similar correlations with radiological progression (construct validity) over 3 years (R = 0.54–0.58; *P *< 0.0001).

**Conclusion:**

APRs add little information on top (and independent) of the combination of clinical variables included in the SDAI. A purely clinical score is a valid measure of disease activity and will have its greatest merits in clinical practice rather than research, where APRs are usually always available. The CDAI may facilitate immediate and consistent treatment decisions and help to improve patient outcomes in the longer term.

## Introduction

Rheumatoid arthritis (RA) is a progressive inflammatory disease, which causes damage and disability [1-5] that can be prevented by promptly initiated and effective therapy [6-9]. To ensure that therapy is effective, frequent clinical assessments are needed [10-12]. For the purpose of disease activity assessment, valid assessment tools using the well established ACR/EULAR/WHO–ILAR (American College of Rheumatology/European League Against Rheumatism/World Health Organization–International League of Associations for Rheumatology) core set variables of disease activity [13-15] are available, such as the Disease Activity Score (DAS) [16]. Also available are the mathematical modifications to the DAS, namely the DAS28 (based on 28-joint counts) and the DAS28-CRP (i.e. the DAS28 using C-reactive protein [CRP] instead of erythrocyte sedimentation rate [ESR]) [17,18], and the recently introduced Simplified Disease Activity Index (SDAI) [19].

However, these scores are rarely used to follow patients in clinical practice because they either employ extensive joint counts (DAS), their computation requires the use of calculators (DAS, DAS28, DAS28-CRP), or their results are not accessible for immediate decision making at the time of patient–physician interaction because of missing laboratory results (DAS, DAS28, DAS28-CRP and SDAI). Although the inclusion of CRP and ESR is fully justified by their face and content validity, the delay associated with their assessment might be one reason why many physicians do not apply composite scores to guide their clinical decisions.

We hypothesized that an abbreviating modification to the SDAI that omits CRP would be a useful score in clinical practice. Our hypothesis was based on the following factors. First, laboratory test results are frequently missing at patient visits, and thus the long-term benefit of a therapeutic approach that is guided by consistent, regular and immediate assessments of disease activity could be jeopardized. Second, simple scores that can be performed 'on the spot' are more likely to be successfully adopted. Third, the principle of numerical summation has been proven and validated to be equivalent to more complex methods of computation [19-23]. Fourth. acute phase reactants (APRs) correlate with each of the other core set variables, especially those employed in the composite indices, suggesting that they may not add importantly to a composite score [24]. Finally, the ACR response criteria consist of an invariable part (joint counts) and a variable part [25], the latter of which employs the APR as one of five measures. Because only three of these measures need to change by more than 20%, the APR is not necessarily required to assess changes in disease activity according to the ACR response criteria; nevertheless, the ACR response criteria agree well with the DAS28 and the SDAI response in data from clinical trials [19,26].

In the present study we established that our initial hypothesis was valid by showing that the contributions made by CRP and ESR to various composite scores are low. We subsequently assessed the correlational, discriminant, and construct validity of a clinical activity index omitting APR in comparison with established scores.

## Method

### Datasets

One source of data employed was a large observational cohort of RA outpatients, who were seen on a regular basis, usually every 3 months. At each visit clinical, functional and laboratory core set variables [18-20] and disease activity according to the composite scores DAS28 and SDAI were documented. Clinical assessments including joint counts were performed by independent, trained assessors who were not involved in treatment decisions. In July 2004, data on 998 patients followed in our clinics had been entered into the database. Each patient's first visit with complete documentation of clinical data was included to assemble the 'routine' cohort. There were 767 patients with at least one complete observation, and the first of these complete observations was used for the analyses. Of all 5070 patient observations that were initially documented, 2564 (50.6%) had missing data. Among these incomplete observations, 45% (*n *= 1150) had missing ESR and/or CRP values.

The second source of data was an independent cohort of newly diagnosed RA patients ('inception' cohort), whose visits were documented in the same manner as described above but starting from their first presentation to the clinic. The referral pattern and detailed follow up of these patients were described elsewhere [9,27]. Radiographs of the hands and feet were obtained every 1–2 years, and were scored using the Larsen method [28] by a team of two experienced readers; they were presented to the readers in chronological order. Reassessment of a random subgroup of 40 radiographs of hands and feet revealed good agreement (R = 0.86, 95% confidence interval [CI] 0.81–0.91). All patients in the inception cohort received disease-modifying antirheumatic drugs, such as methotrexate, as soon as the diagnosis was made, with a few exceptions in patients who refused to take such therapy immediately.

The demographic and disease activity characteristics of patients in both cohorts are summarized in Table 1. Because several baseline variables were not normally distributed (see below), we present the median along with the first and third quartiles as robust descriptive measures.

### Distribution of study variables and appropriateness of test statistics

Whenever variables were normally distributed, as assessed using the Kolmogorov–Smirnov test, we performed parametric test statistics (such as Pearson correlation, or one-way analysis of variance [ANOVA]). In several cases, skewed distributions required the use of nonparametric tests (such as Spearman rank correlation). However, the exploratory analysis on the contribution of APRs to the various composite scores was based on a linear regression model despite non-normal distributions of several variables, given the large numbers of observations in the routine cohort (*n *= 767; Table 1), which is sufficient to invoke the central limit theorem.

### Analysis of the contributions of acute phase reactants to current composite scores

Calculations of the DAS28 and SDAI are based on the following: numbers of swollen and tender joints (swollen joint count [SJC] and tender joint count [TJC]), employing the 28 joint count; evaluator's and/or patient's global assessment of disease activity (EGA, PGA); and CRP or ESR. The following formulae are the basis for their calculation [16,19]:

DAS28 = (0.56 × TJC^1/2^) + (0.28 × SJC^1/2^) + (0.7 × ln [ESR]) + (0.014 × PGA [in mm])

SDAI = SJC + TJC + PGA (visual–analogue scale [VAS; in cm]) + EGA (VAS [in cm]) + CRP (in mg/dl)

In addition, we calculated a version of the DAS28 that, like the SDAI, employs CRP rather than ESR, and is obtained as follows [18]:

DAS28-CRP = (0.56 × TJC^1/2^) + (0.28 × SJC^1/2^) + (0.36 × ln [CRP; in mg/l])+1) + (0.014 × PGA [in mm]) + 0.96

To determine whether our clinical hypothesis that CRP makes a small contribution to the SDAI would withstand statistical analysis, we first evaluated the contributions made by individual component variables to the SDAI. We constructed a perfect fit regression model to predict the score by its items, using cross-sectional patient observations from the routine dataset (*n *= 767). For each variable contained in the SDAI, we determined the contribution made to the SDAI (R^2^) when the variable was introduced as first (zero order) or last (final) variable in the model. In addition, we determined each item's colinearity, presented as the proportion of its variance that was explained by the other items in the score, which equals the term (1 - tolerance) × 100. The three parameters (zero order and final model contributions, and colinearity diagnostics) provide overlapping information, and were used to assess the statistical characteristics of CRP in the SDAI.

We then followed an analogous sequence of analyses to determine model contribution and colinearity of individual component variables to the DAS28 and the DAS28-CRP. To allow construction of the perfect fit model, introduction of items into the regression model employed transformed values according to the respective formulae (SJC and TJC as square roots, and ESR and CRP as their natural logs).

### Clinical activity index and assessment of comparative validity

Next, we calculated the Clinical Disease Activity Index (CDAI) as follows:

CDAI = SJC + TJC + PGA (in cm) + EGA (in cm)

We determined several aspects of validity of the CDAI [29]: correlational validity refers to comparison with other measures of disease activity; discriminant validity in this setting relates to the correlation of changes in the scale with changes in other measures of disease activity; and construct validity considers correlation with important outcomes of the disease, such as radiological progression.

#### Correlational validity

Correlational validity between CDAI, DAS28 and SDAI was assessed in patients from the routine cohort (*n *= 767) using Spearman's rank statistics. In addition, we calculated 95% CIs using Fisher's approximation. Next, we used the Health Assessment Questionnaire Disability Index (HAQ) score as an additional comparator in the correlation analysis with these indices (*n *= 720). As a functional measurement, the HAQ is determined by accumulated joint damage but also by disease activity [30-32]. Moreover, the HAQ is an independent comparator that does not include joint counts, global assessments, or APRs, in contrast to composite scores, which are all based on similar sets of variables. We then validated these results in an independent group of patients at their first presentation using the inception cohort (*n *= 105). In this manner, the results from a cohort with, on average, moderate disease activity were validated in another one with high disease activity (Table 1).

In addition to the presented correlation coefficients, we sought to determine the agreement of the different scores in individual patients. We therefore created 10 patient groups of equal size based on the patients' DAS28 ranks within the cohort. The groups were ordered (i.e. the first group comprised the 10% of patients with the lowest DAS28, and the last group comprised the 10% with the highest DAS28 values). Then, we grouped the patients in the same way based on their CDAI, SDAI and DAS28-CRP ranks. Based on these groups, we used weighted kappa statistics to assess agreement of different scores on individual patients.

#### Discriminant validity

For the assessment of discriminant validity we characterized patients by their degree of improvement according to the ACR response criteria within 1 year after entering the inception cohort (*n *= 91 with complete baseline and 1 year data). We divided ACR responses into three groups: lack of response (<20% improvement by ACR response criteria), major response (≥ 70% improvement), or moderate response (≥ 20% but <70% improvement). Using one-way ANOVA, we analyzed whether changes in the various continuous scores were greater in higher ACR responder groups, and whether these differences were statistically significant at the group level. We then used *post hoc *t-tests with Bonferroni adjustment to determine which groups were statistically different in pairwise comparisons. Also, effect sizes for each group were calculated as changes in scores divided by their baseline standard deviations [33].

In addition to the comparison with ACR response, we correlated changes in the continuous scores with respective changes in HAQ scores during the first year of disease (*n *= 91), using Spearman rank correlation and Fisher's approximation as described above. However, in this early cohort, HAQ score mainly reflects disease activity rather than being a measure of functional outcome (i.e. a surrogate for construct validity).

#### Construct validity

Radiographic data were available for the majority of the 80 patients in the inception cohort who were followed for 3 years (*n *= 56), which constituted a clinically meaningful time frame in which to detect major changes in damage. We performed linear (Pearson) correlation of time averaged disease activity (equivalent to area under the curve) for DAS28, SDAI and CDAI with changes in Larsen score over 3 years. Again, we calculated 95% CIs as above. For simplicity, we did not employ more sophisticated methods, such as longitudinal modelling (e.g. by generalized estimating equations), in this validation analysis.

## Results

### Contribution of acute phase reactants to composite scores

**Figure 1:**
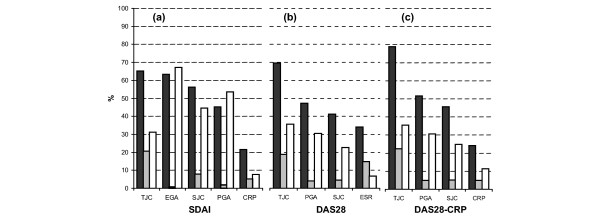
**Contribution of individual variables to composite scores**. Explanation of score variability for **(a) **the Simplified Disease Activity Index (SDAI), **(b) **the Disease Activity Score (DAS)28, and **(c) **the DAS28-CRP for the respective clinical and acute phase reactant (APR) variables, at zero-order (i.e. R^2 ^if the variable was introduced as the first one; black bars) or finally (i.e. R^2 ^if variable was introduced in the model as the last one; grey bars), and item colinearity within the respective composite index (1 - tolerance, expressed as percentage; white bars; *n *= 767). CRP, C-reactive protein; ESR, erythrocyte sedimentation rate; PGA/EGA, patient/evaluator global assessment of disease activity (100 mm visual analogue scale); TJC/SJC, tender/swollen joint count (28 joints).

Figure 1 depicts the results from the perfect fit regression models. Items are ordered according to their contribution when introduced into the model as first variable (zero-order R^2 ^contribution; dark bars); the independent variables that best accounted for the SDAI (Fig. 1a) were TJC (R^2 ^= 65.1%) and EGA (R^2 ^= 63.4%), and the variable with the smallest R^2 ^was CRP (21.5%). When individual variables were introduced as last items into the model (final R^2^; grey bars), the contribution was least for EGA (0.7%) and PGA (1.8%), according to their colinearity (>50% for each). The final R^2 ^for CRP was only 5.1%, despite the practical absence of colinearity (7.7%). These analyses indicate that CRP was adding independent information to the score (low colinearity), but that its changes were not likely to be substantially reflected in changes in the SDAI (low model contribution).

The results of analogous analyses for the DAS28 and its items are shown in Fig. 1b. Similar to the results of the SDAI analysis, ESR (as the APR) made the smallest independent contribution to the DAS28 (R^2 ^= 34.0%), and the smallest colinearity (6.6%), although the final contribution was somewhat higher (14.8%). As in the SDAI, there was a significant level of colinearity of residual items (white bars), but to a somewhat lesser degree.

To determine whether the difference in final contributions between ESR in the DAS28 and CRP in the SDAI (14.8% versus 5.1%), given similar degrees of colinearity (6.6% versus 7.7%), was score related (i.e. DAS28 versus SDAI) or item related (i.e. ESR versus CRP), we analyzed the newly proposed modification to the DAS28 [18], which includes CRP instead of ESR but otherwise identical variables (DAS28-CRP; Fig. 1c). Here, despite the differences in construction of and component weighing in the two scores, the contributions made by CRP (zero order 24.5%, final 4.8%) reached similar levels to CRP in the SDAI (21.5% and 5.1%, respectively). The low colinearity of APRs in all three scores indicates that they provide information distinct from the clinical measures. However, the low model contribution of CRP (about 5%) indicates that only a very small proportion of variance in the respective indices remains unexplained without CRP, which is in accord with the small numerical value of CRP in the SDAI and the DAS28-CRP. Likewise, ESR made a relatively low model contribution to the DAS28, which is line with a significant correlation between these two APRs in the studied cohort (R = 0.63; *P *< 0.001). hypothesized that APRs make limited contribution to the SDAI

Because our initial hypothesis – that CRP makes a limited contribution to the SDAI, and that excluding CRP from the SDAI will yield a simple and immediately calculable score – was supported by these statistical analyses, we next validated the CDAI using the cross-sectional 'routine' cohort and the independent, longitudinal inception cohort of patients with RA. The quartiles and ranges for the CDAI and for all other mentioned scores are shown in Table 2 for both patient cohorts.

### Cross-sectional correlation and validation of composite scores and Health Assessment Questionnaire disability index

**Figure 2:**
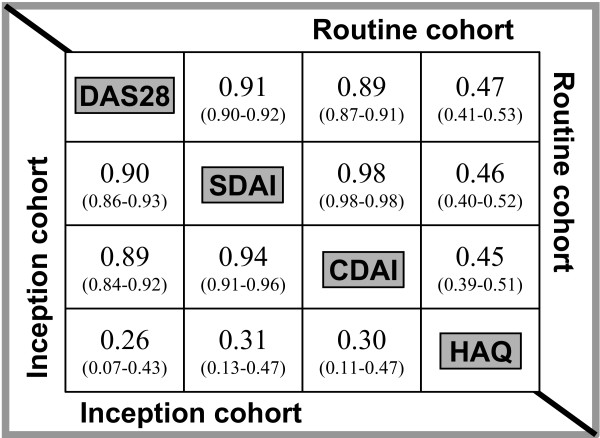
**Cross-sectional correlation of composite scores and correlation with HAQ scores**. Matrix displaying Spearman rank coefficients (95% confidence intervals) for cross-sectional correlations of Disease Activity Score (DAS)28, Simplified Disease Activity Index (SDAI), Clinical Disease Activity Index (CDAI), and Health Assessment Questionnaire (HAQ) in the routine cohort (upper diagonal half; *n *= 720 for correlations with HAQ, otherwise *n *= 767) and the inception cohort (lower diagonal half; *n *= 104 for correlation with HAQ, otherwise *n *= 105).

We next analyzed the correlation between the DAS28, SDAI and CDAI, as well as the correlation between these scores and the HAQ disability index in the routine cohort, which revealed similar correlation coefficients for CDAI and SDAI when compared with DAS28 (Fig. 2, upper diagonal half; *n *= 767). This correlation was fully validated by virtually identical coefficients obtained in the analysis of the inception cohort (Fig. 2, lower diagonal half), in which patients had higher disease activity. Likewise, Spearman rank correlations with the HAQ revealed comparable results for DAS28, SDAI and CDAI within each of the patient cohorts. The comparable correlation coefficients obtained for all three scores in two independent cohorts strengthens the results obtained from the cross-sectional analysis, because they were not influenced by the level of disease activity, the patients' disease duration, or treatment status, which were all different between patients in the routine and those in the inception cohort. Although there were differences in the degree of correlation with the HAQ between the two cohorts, this pertained to all three disease activity scores in a similar manner.

In a further analysis, based on the cohort ranks of each patient's DAS28, DAS28-CRP, SDAI and CDAI values, we divided the patients into 10 ordered groups for each of the four scores (from the group comprising the 10% of patients with the lowest activity to that consisting of the 10% with the highest activity, by respective score). We then analyzed the agreement of these categorizations between scores using weighted kappa statistics [34]. Kappa values range from 0 (agreement as expected by chance) to 1 (maximum possible agreement beyond chance). For this analysis of individual patient allocation into the different groups, there was good agreement of the CDAI with the DAS28-CRP and the DAS28 (κ = 0.79 and 0.70, respectively). The results were similar when the DAS28 and its derivative, the DAS28-CRP, were compared (κ = 0.80). Not surprisingly, there was excellent agreement between CDAI and SDAI (κ = 0.89).

### Changes in composite scores in relation to American College of Rheumatology response and to changes in Health Assessment Questionnaire scores

**Figure 3:**
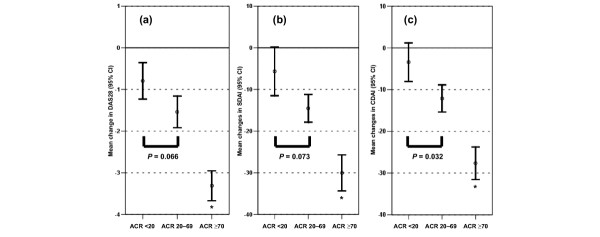
**Changes in composite scores in relation to ACR response**. Changes in **(a) **Disease Activity Score (DAS)28, **(b) **Simplified Disease Activity Index (SDAI) and **(c) **Clinical Disease Activity Index (CDAI) in relation to the achieved American College of Rheumatology (ACR) response of 91 patients in the inception cohort. ACR ranges were defined as ACR <20 (*n *= 28, 30.8%), ACR 20–69 (*n *= 20, 22.0%) and ACR ≥ 70 (*n *= 43, 47.2%), allowing analysis of independent observations. Error bars span the 95% confidence interval of the mean. Differences in group changes were statistically significant for all three scores (*P *< 0.0001, one-way analysis of variance). Presented *P *values for *post hoc *pairwise group comparisons are subjected to Bonferroni adjustment. *P < 0.0001 for ≥ ACR70 group compared with other groups.

In the inception cohort, ACR20 responses were achieved by 69% of patients at the end of the first year, ACR50 by 59%, and ACR70 by 47%. To allow comparison of changes in composite scores in individuals with ACR responses, we grouped patients' improvements into the following categories: non-response (ACR response <20%; *n *= 28, 30.8%), moderate response (20–69% improvement; *n *= 20, 22.0%) and major response (≥ 70% improvement; *n *= 43, 47.3%). The high rate of ACR70 responders in this clinic cohort treated with traditional disease-modifying antirheumatic drugs is in accordance with previous observations in similar patient cohorts [12,35]. At the group level, score responses of the DAS28 (Fig. 3a), SDAI (Fig. 3b) and CDAI (Fig. 3c) increased with respect to the ACR response categories (*P *< 0.0001, one-way ANOVA). *Post hoc *Bonferroni-adjusted pairwise t-tests revealed significant differences for the comparison of the ACR ≥ 70% responders with the other groups (*P *< 0.0001). The ACR <20 and ACR 20–69 groups were statistically different only in the CDAI analysis (*P *= 0.032; Fig. 3c). These findings indicate that, at the group level, the DAS28, SDAI and CDAI were sensitive in discriminating between different response categories. This is further supported by calculating the effect size for the three scores after 1 year of observation: for the DAS28 the effect size in the ACR20–69 responders was 2.4 times higher than in the ACR nonresponders; likewise, the effect size of the ACR70 responders was 4.4 times that in the nonresponders. The same analyses revealed an effect size increases of 2.7-fold and 4.1-fold, respectively, for the SDAI, and of 3.3-fold and 6.5-fold, respectively, for the CDAI. Thus, using effect size calculations, all three scores discriminated various degrees of ACR responsiveness from ACR nonresponsiveness to a similar extent. Also, the 1 year changes in the HAQ in these 91 patients were similarly correlated with the 1 year changes in all three scores: for DAS28, R = 0.32 (95% CI 0.12–0.49; *P *= 0.001); for SDAI, R = 0.38 (95% CI 0.19–0.54; *P *< 0.001); and for CDAI, R = 0.39 (95% CI 0.20–0.55; *P *< 0.001).

### Radiological outcome

**Figure 4:**
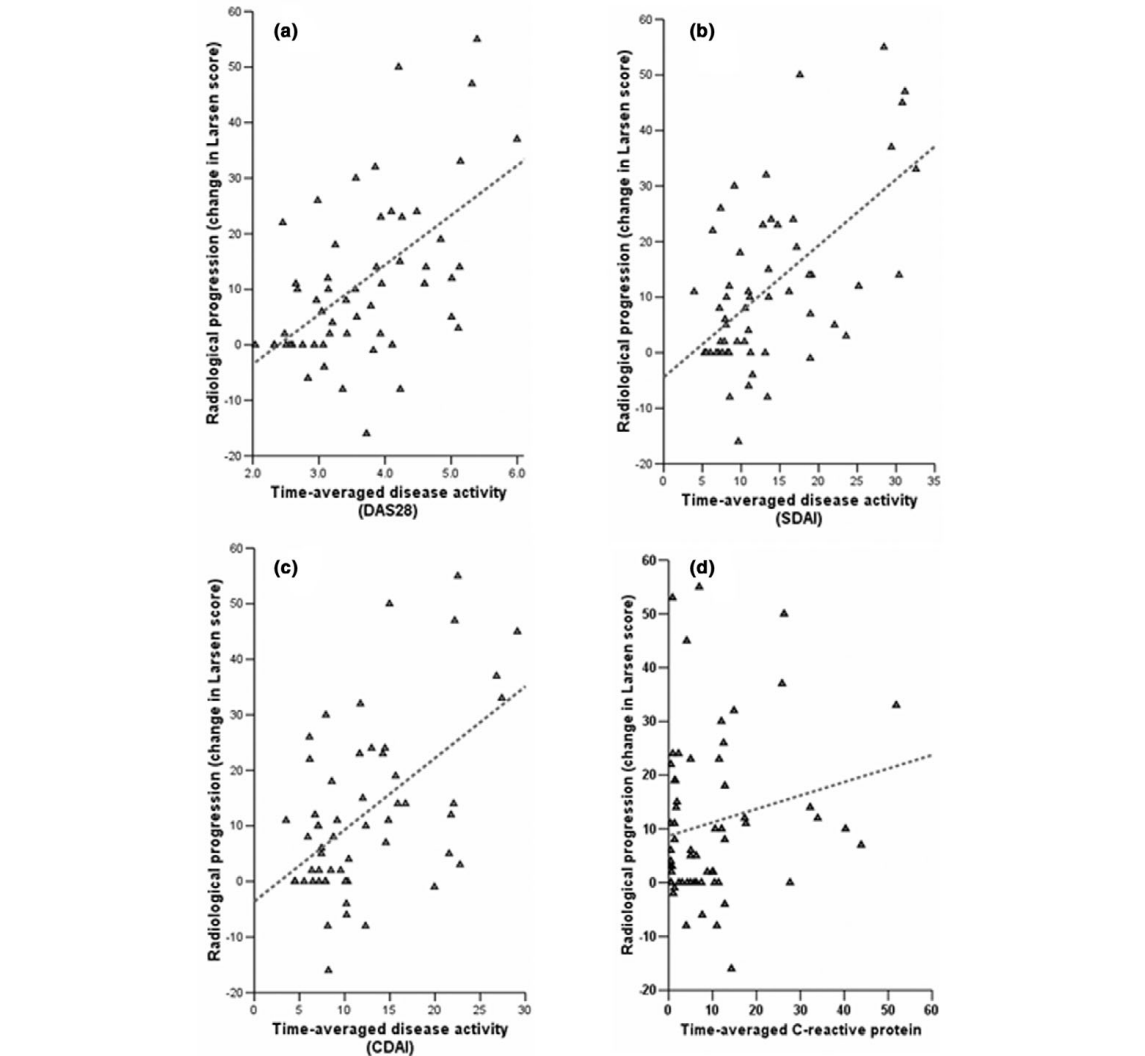
**Association of composite scores with radiological outcome**. Correlation with changes in Larsen scores within 3 years from entering the inception cohort (*n *= 56) of time-averaged **(a) **Disease Activity Score (DAS)28 (R = 0.58, 95% confidence interval [CI] 0.37–0.73), **(b) **Simplified Disease Activity Index (SDAI; R = 0.59, 95% CI 0.39–0.74), and **(c) **Clinical Disease Activity Index (CDAI; R = 0.54, 95% CI 0.32–0.70). All correlations are significant (*P *< 0.0001). **(d) **C-rectaive protein (CRP; R = 0.28, 95% CI 0.02 to 0.51; *P *= 0.025).

To compare construct validity between the composite scores, we performed a linear correlation analysis between time-averaged DAS28, SDAI, CDAI and changes in Larsen scores over 3 years (*n *= 56). The R coefficients were 0.58 (95% CI 0.37–0.73), 0.59 (95% CI 0.39–0.74) and 0.54 (95% CI 0.32–0.70), respectively. All correlations were significant (*P *< 0.0001). Figure 4a–c permits visual judgement of this relationship for each score, and a line of best fit has been added based on the given observations. Moreover, there was significant correlation between time integrated CRP with changes in Larsen scores (Fig. 4d), as was previously reported by others [36-38].

## Discussion

In this study we showed that the CDAI, a simple composite index obtained by numerical summation of four solely clinical variables, is a valid instrument with which to follow patients with RA. Our hypothesis was originally based on feasibility arguments, namely the frequent lack of immediate access to laboratory results in the clinic, but was further strengthened by statistical arguments related to the low contribution made by the acute phase response to the composite scores. In fact, all data obtained support our clinically derived hypothesis that APRs provide little information on actual disease activity on top of that provided by the combination of several clinical components. This was the case for all analyzed RA activity scores, despite the differences in their construction and component weighing.

For many rheumatologists, this lack of additional information provided by APR may be intriguing because CRP and ESR are among the most commonly used laboratory tests in the evaluation of RA disease activity [39], and their importance as surrogates of the disease process, as well as predictors of disease outcome, are well recognized and irrefutable [36-38]. However, APRs did not seem to contribute information to composite scores that was sufficiently important to change judgement of disease activity, in addition to merely using clinical measures. In fact, when we divided all patients into 10 groups based on their disease activity ranks within the cohort, as measured using the different scores, we found statistical agreement that was indicative of high clinical conformity of classifications by different scores. All of these findings indicate that content validity of the CDAI is well maintained despite the absence of CRP as a component.

In accordance with these notions is the observation that as much as 85% of the variance in the DAS28 was explained without ESR; 95% of the variances in the SDAI and the DAS28-CRP were explained by their composing clinical variables (i.e. without CRP). The similarity in these results between the DAS28-CRP and the SDAI further supports previous indications that transformation and/or weighing of the clinical variables does not confer an advantage compared with their simple numerical summation [21-23,40]. However, it should be borne in mind that the DAS28-CRP has only recently been made public and must be regarded with caution until it has been more widely studied; in fact, the present investigation may represent the first validation of the DAS28-CRP. Interestingly, our analyses reveal a high degree of colinearity between the two global assessments employed in the SDAI and CDAI. Because both patient and physician global assessment are parts of the widely applied and validated ACR/EULAR/WHO-ILAR core set variables of RA disease activity assessment, it would not be intuitive to eliminate any one of them, especially as, in contrast to the APRs, they do not correlate with structural damage independently. In addition, because these two variables are usually assessed jointly, the elimination of any one of them would not increase the feasibility of calculating the score.

In a cross-sectional analysis of a large number of patients, the CDAI not only had correlational validity compared with the SDAI, from which it was derived, but also compared with the DAS28 and the DAS28-CRP. Also, there was no difference beyond chance in the correlation of CDAI with the HAQ as compared with the respective correlations of SDAI and DAS28 with the HAQ. This finding is especially noteworthy because the HAQ is a functional measure, which is not based on or constructed with core set elements used in the DAS28 or SDAI. Moreover, when related to different degrees of ACR response, the results obtained using CDAI were graded with statistically significant and clinically meaningful differences between all group means, and were very similar to those seen for the respective DAS28 and SDAI groups. Also, for the CDAI, effect sizes appeared to be even more graded between the different ACR responder groups.

Thus, although none of the comparators in this study represents a 'gold standard' for disease activity measurement, the validity of the new score was proven not only with respect to other composite scores but also with respect to the HAQ, which is a completely distinct construct. In addition, the CDAI was shown to have very good agreement with other composite indices on the categorization of individual patients, which is an important aspect in the clinical use of this score. Furthermore, all mentioned correlation analyses were successfully validated in a second, completely independent cohort of newly diagnosed patients with RA who overall had a higher level of disease activity and were untreated at baseline. The different characteristics of the two cohorts, and the similar correlation coefficients for the three indices obtained within each cohort indicate that the application of our findings might not be confined to patient cohorts with particular characteristics, such as disease duration or disease activity.

A limitation of the CDAI is that many physicians do not perform detailed joint counts in the assessment of RA disease activity [38]. On the other hand, joint counts are also required for other composite disease activity scores, and the CDAI allows elimination of at least one variable that is frequently missed at patient visits – the APR. Although a considerable number of measurements was missing in the overall source dataset, these missing data were random. This was also evident from the similar clinical characteristics of patients with and without available APR measurements. Therefore, and given the large number of complete patient observations, an unbiased analysis was assured. Like for the DAS28 [41], a possible criticism of the CDAI is that it does not include assessment of joints in the feet; however, in the course of proving the reliability of the 28 joint count [42,43], it was found that this reduced joint count reflects overall joint involvement very well and that, in the presence of low joint counts, the joints of the feet rarely add a significant number of additionally involved joints – a finding that we have also observed in our database (data not shown). It might also be regarded as a further limitation that the CDAI was not developed by factor and/or discriminant analysis of individual variables. However, the value of all core set variables has been shown repeatedly [10-12] and their responsiveness has likewise been demonstrated [26]. In addition, there are several conceptual and methodological advantages of composite scores compared with individual items [29]. Moreover, the SDAI, from which the CDAI was derived, has also been validated and shown to have practicability, discriminant capacity and sensitivity to change in several studies [21-23,37]. Likewise, as a composite score, the test–retest reliability of the CDAI is based on the reliability of its individual components, which, although not assessed here, has proven to be good.

Despite omission of the APR from the formula, the CDAI maintained a clinically important ability to predict outcome, measured as radiological progression over 3 years. This stability of construct validity across the scores is therefore also in accord with our initial hypothesis. Interestingly, the deletion of the APR from the SDAI did not change the correlation of the score with radiographic progression; there was a similar degree of correlation with radiographic changes whether DAS28 (using ESR), SDAI (using CRP), or CDAI (using no APR) were employed. Furthermore, the observation that the APR alone also was associated with radiographic progression is not only in accordance with previous reports [36-38] but also suggests an independent relationship with structural damage of both clinical variables (as reflected by the composite CDAI score) and APR. We did not use HAQ scores as an outcome measure because in this early cohort the links between damage and function are expected to be small [31,44]. HAQ scores in this cohort would therefore be a surrogate of disease activity, rather than an independent measure of irreversible loss of function [30,31].

Our introduction of the CDAI was not intended to suggest that the acute phase response does not represent an important measure in the follow up of RA, or that it should be deleted from existing indices such as the DAS28 and the SDAI. In particular, the ESR contributes 15% to the DAS28 composition, which is not an irrelevant amount of information. However, the validity of the CDAI, as revealed here by multiple statistical analyses in two different cohorts, shows that the APR is not an absolute requirement in the context of disease activity scores. In fact, we would urge physicians to continue to obtain an APR measure regularly during follow up because, like the CDAI, it reflects disease activity and correlates with long-term outcome. However, as stated above, the APR can be employed as an independent measure as well as being a part of a composite index.

Because calculation of the SDAI (and of the DAS28) is frequently limited at the time of the patient's visit either by a wait for laboratory results or their unavailability, omitting the APR from the score allows unlimited and immediate assessment of disease activity by including only variables that are available by physical examination and patient questioning at the time of interaction with the patient. Therapeutic decisions will then be possible without further delay. Of course, clinic settings can be revised to have laboratory results delivered at the time of patient visits, although this may not be easy in all situations, and in reality is often not the case. Thus, using a purely clinical score facilitates consistent patient assessment, which might be more attractive for routine application to many physicians, who currently base their treatment decisions on more general and subjective impressions rather than on standardized assessments. The fact that joint counts are frequently not assessed routinely does not diminish these notions; deletion of joint counts from composite scores cannot be justified for a disease of the joints, and joint counts can always be performed at the time of patient visit to the clinic by the physician or another assessor. Moreover, in this age of expensive therapies, consistent assessment of disease activity might soon become compulsory from the payer's perspective. Thus, the ability to adopt a simple but valid score will potentially have great implications with respect to implementation of new therapeutic concepts. At the same time, less frequent laboratory investigations do not appear to impair the physician's ability to detect adverse treatment effects, but can reduce the overall costs of care considerably [45-47].

Of course, further validation of the CDAI will be required to fully confirm its value. Such additional investigations should include analyses of construct validity with regard to radiographic damage and predictive value with regard to long-term functional outcome in larger cohorts of patients. In addition, cutoffs for disease activity categories, including remission, as well as changes that reflect important responses must be determined. Such analyses are currently underway.

## Conclusion

Our findings indicate that the CDAI – a composite score that employs only clinical variables and omits assessment of an APR – has similar validity to other currently employed composite indices for following patients with RA. Also, using numerical summation, this score is very easy to calculate. For these reasons, the CDAI should facilitate decision making by physicians and avoid lags in efficient treatment adaptation for patients with RA. According to current knowledge, such intensified and prompt patient care can be expected to reduce the individual [12,48] and socioeconomic impact of the disease in the longer term.

## Abbreviations

ACR = American College of Rheumatology; ANOVA = analysis of variance; APR = acute phase reactant; CDAI = Clinical Disease Activity Index; CI = confidence interval; CRP = C-reactive protein; DAS = Disease Activity Score; ESR = erythrocyte sedimentation rate; EULAR = European League Against Rheumatism; HAQ = Health Assessment Questionnaire Disability Index; EGA = evaluator global assessment; PGA= patient global assessment; RA = rheumatoid arthritis; SDAI = Simplified Disease Activity Index; SJC = swollen joint count; TJC = tender joint count; VAS = visual–analogue scale (100 mm); WHO–ILAR = World Health Organization–International League of Associations for Rheumatology.

## Figures and Tables

**Table 1:** Characteristics of patients in routine and inception cohorts

Characteristic	Routine cohort (cross-sectional)	Inception cohort (longitudinal)
Patients (*n*)	767	106
Age (years; mean ± SD)	54.1 ± 14.9	50.5 ± 15.6
Sex (% female)	79.9	75.2
Rheumatoid factor (% positive)	55.3	78.1
Disease duration at baseline (mean ± SD)	8.1 ± 10.6 years	11.5 ± 12.5 weeks
Duration of follow up (years; mean ± SD; range)	-	3.2 ± 1.3; 1–7.25
Disease activity characteristics (median [1st;3rd quartile])	At cross-section	At baseline
Swollen joint count (0–28)	3 (1;7)	7 (4;13)
Tender joint count (0–28)	2 (0;6)	8 (3;16)
ESR (mm; normal <20)	23 (14;55)	49 (24;70)
CRP (mg/dl; normal <1.0)	1.1 (0.5;2.7)	5.1 (1.9;17.0)
Patient assessment of pain (mm; 0–100)	37 (19;53)	50 (32;66)
Patient global assessment of activity (mm; 0–100)	37 (18;58)	51 (33;66)
Evaluator global assessment of activity (mm; 0–100)	34 (19;49)	44 (31;58)
HAQ (0–3)	0.875 (0.25;1.5)	0.75 (0;1.5)
Larsen score	-	1 (0;7)
Completeness of data for analysis		
Cross-sectional correlation between composite indices (*n *[%])	767/767 (100)^a^	105/106 (99.1%)^b^
Cross-sectional correlation with HAQ scores (*n *[%])	720/767 (93.9)	104/106 (98.1)^b^
Discriminant validity, 1-year follow up (*n *[%])	-	91/100 (91.0%)
Construct validity, 3-year follow up^c ^(*n *[%])	-	56/80 (70.0%)

^a^Completeness of data was the prerequisite for inclusion. ^b^Used to validate the results from the cross-sectional analyses in the routine cohort. ^c^Including complete radiological data. CRP, C-reactive protein; ESR, erythrocyte sedimentation rate; HAQ, Health Assessment Questionnaire; SD, standard deviation.

**Table 2:** Values of composite indices in the two cohorts.

Composite scores (range^a^)	Routine cohort (*n *= 767)			Inception cohort (*n *= 105)		
	
	Median	1st;3rd Quartile	Range	Median	1st;3rd Quartile	Range
DAS28 (0.5–9.1)	4.09	2.99;5.17	0.50–8.56	5.62	4.81;6.44	2.84–8.28
DAS28-CRP (1.0–8.5)	3.78	2.71;4.82	1.60–8.28	4.67	4.04;5.50	2.35–7.42
SDAI (0–86)	16.7	8.1;26.7	0.5–78.9	29.0	20.1;41.6	7.5–77.0
CDAI (0–76)	14.8	6.5;23.3	0–67.8	25.6	17.1;37.9	6.3–70.2

^a^Maximum possible ranges of acute phase reactants assumed: 5–100 mm for erythrocyte sedimentation rate; 0–10 mg/dl for C-reactive protein (CRP). CDAI, Clinical Disease Activity Index; DAS, Disease Activity Score; SDAI, Simplified Disease Activity Index.
